# Cellular and Deafness Mechanisms Underlying Connexin Mutation-Induced Hearing Loss – A Common Hereditary Deafness

**DOI:** 10.3389/fncel.2015.00202

**Published:** 2015-05-29

**Authors:** Jeffrey C. Wingard, Hong-Bo Zhao

**Affiliations:** ^1^Department of Otolaryngology, University of Kentucky Medical Center, Lexington, KY, USA

**Keywords:** gap junction, non-syndromic hearing loss, cochlear supporting cell, hair cell, cochlear development, active cochlear amplification, potassium recycling, inner ear

## Abstract

Hearing loss due to mutations in the connexin gene family, which encodes gap junctional proteins, is a common form of hereditary deafness. In particular, connexin 26 (Cx26, *GJB2*) mutations are responsible for ~50% of non-syndromic hearing loss, which is the highest incidence of genetic disease. In the clinic, Cx26 mutations cause various auditory phenotypes ranging from profound congenital deafness at birth to mild, progressive hearing loss in late childhood. Recent experiments demonstrate that congenital deafness mainly results from cochlear developmental disorders rather than hair cell degeneration and endocochlear potential reduction, while late-onset hearing loss results from reduction of active cochlear amplification, even though cochlear hair cells have no connexin expression. However, there is no apparent, demonstrable relationship between specific changes in connexin (channel) functions and the phenotypes of mutation-induced hearing loss. Moreover, new experiments further demonstrate that the hypothesized K^+^-recycling disruption is not a principal deafness mechanism for connexin deficiency induced hearing loss. Cx30 (*GJB6*), Cx29 (*GJC3*), Cx31 (*GJB3*), and Cx43 (*GJA1*) mutations can also cause hearing loss with distinct pathological changes in the cochlea. These new studies provide invaluable information about deafness mechanisms underlying connexin mutation-induced hearing loss and also provide important information for developing new protective and therapeutic strategies for this common deafness. However, the detailed cellular mechanisms underlying these pathological changes remain unclear. Also, little is known about specific mutation-induced pathological changes *in vivo* and little information is available for humans. Such further studies are urgently required.

## Introduction

Gap junctions are intercellular channels that connect the cytoplasm of adjacent cells, providing a direct intracellular conduit for intercellular communication. A gap junctional channel is formed by two hemichannels; each hemichannel is composed of six subunits (Bennett et al., 1991; Harris, 2001). Gap junctions exist in both vertebrates and invertebrates. The gap junction proteins in vertebrates are mainly encoded by the connexin gene family, which consists of more than 20 connexin isoforms (Willecke et al., 2002), whereas the gap junction proteins in invertebrates are encoded by the unrelated innexin family. Pannexin is an innexin homologous gene and also encodes gap junctional proteins in vertebrates (Bruzzone et al., 2003a; Baranova et al., 2004). However, unlike connexins, pannexins usually form non-junctional membrane channels on the cell surface to provide an intracellular–extracellular conduit and are considered unable to form integral gap junctional channels between cells (Boassa et al., 2007; Penuela et al., 2007; Sosinsky et al., 2011).

Unlike typical ion channels (such as K^+^, Na^+^, and Ca^++^ channels), gap junction channels possess a relatively large pore size (~10–15 Å) and can allow passage of ions, cell signaling molecules, and small molecules up to ~1.5 kDa (Bennett et al., 1991; Harris, 2001). This direct intercellular communication pathway plays an important role in embryonic and postembryonic development, cancer suppression, and many physiological and pathological functions. Connexin mutations can cause severe deafness and are responsible for most cases (> 50%) of hereditary hearing loss in the clinic (Kelsell et al., 1997; Zelante et al., 1997; Denoyelle et al., 1998; Estivill et al., 1998; Kelley et al., 1998), indicating that connexin gap junctions play a critical role in hearing. Connexin mutations, mutation-induced auditory phenotypes, and gap junctional function in the cochlea have been extensively summarized by previous reviews [e.g., Zhao et al. (2006), Castillo and Castillo (2011), and Chan and Chang (2014)]. In this review, we mainly focus on the pathogenesis and deafness mechanisms underlying this common hereditary deafness.

## Phenotypes of Cx26 (GJB2) Mutation-Induced Hearing Loss

Connexin 26 (Cx26, *GJB2*) mutations are a common genetic cause for non-syndromic hearing loss and are responsible for ~50% of non-syndromic hearing loss in children (Rabionet et al., 2000). Non-syndromic deafness can be autosomal dominant deafness (DFNA, DFN: deafness; A: dominant), autosomal recessive deafness (DFNB, B: recessive), or X-linked deafness (DFNX, X: X-linked). Each type is numbered in the order in which it was described. The majority of Cx26 mutations are recessive (DFNB1) but a few are dominant (DFNA3). In the clinic, various symptoms and auditory phenotypes are observed. Cx26 mutations can result in a mild-moderate to profound sensorineural hearing loss (Zhao et al., 2006; Castillo and Castillo, 2011; Chan and Chang, 2014). The mutation-induced hearing loss is not always congenital and can be late-onset and progressive, starting or occurring in childhood (Orzan and Murgia, 2007; Pollak et al., 2007; Gopalarao et al., 2008; Chan and Chang, 2014). This diversity of clinical appearances implies that hearing loss induced by Cx26 mutations has various pathological changes and different underlying deafness mechanisms.

## Functional Analyses of Cx26 Deafness Mutations

To date, more than 100 *GJB2* mutations have been identified to be associated with deafness [connexins and deafness Web site: http://davinci.crg.es/deafness/index.php, and also see Mani et al. (2009), Castillo and Castillo (2011), and Chan and Chang (2014)]. Functional analyses in transfected cells *in vitro* reveal a variety of pathogenic changes caused by these deafness mutations. (i) Most mutants cannot correctly track to the cell surface to form functional gap junction channels (Martin et al., 1999; Choung et al., 2002; D’Andrea et al., 2002; Thonnissen et al., 2002; Bruzzone et al., 2003b; Wang et al., 2003; Bicego et al., 2006). (ii) Mutants can also have dominant or trans-dominant negative effects on wild-type (WT) Cx26 and co-expressed Cx30 (Thomas et al., 2004; Bicego et al., 2006; Deng et al., 2006; Palmada et al., 2006; de Zwart-Storm et al., 2008; Yum et al., 2010; Zhang et al., 2011). (iii) Some mutants (e.g., p.M34T) can be correctly synthesized and target to the plasma membrane, but cannot form efficient intercellular gap junction channels (Skerrett et al., 2004; Bicego et al., 2006). (iv) Some mutants retain permeability to ions but not to small molecules. For example, the Cx26 p.V84L mutant is permeable to ions but is impermeable to IP_3_ (Beltramello et al., 2005; Zhang et al., 2005). (v) Some deafness-associated mutants, such as p.V84L and p.V95M, can form functional homotypic gap junction channels but cannot form functional heterotypic channels (Choung et al., 2002; Thonnissen et al., 2002; Bruzzone et al., 2003b; Wang et al., 2003), indicating that these mutants may specifically impair heterogeneous channels *in vivo*. A gap junction is formed by two hemichannels. Hemichannels can also solely function on the cell surface to provide an intracellular–extracellular conduit. Connexin hemichannels are usually closed at normal extracellular Ca^++^ level under normal physiological conditions but could be opened under some pathological conditions (Bennett et al., 2003; Goodenough and Paul, 2003). (vi) Some mutants, e.g., p.G45E, can cause abnormal hemichannel activity and opening at the normal extracellular Ca^++^ level leading to cell lysis and death (Stong et al., 2006; Gerido et al., 2007). (vii) Finally, it has been reported that the Cx26 p.R75W mutant can impair gap junctional plaque formation and reduce the area of gap junctional plaque and protein levels. The reduction is associated with excessive endocytosis with increased expression of caveolin 1 and 2 (Kamiya et al., 2014). However, except for a few mutations, there is no apparent, demonstrable relationship between mutation phenotypes and auditory phenotypes. There is also no definite relationship between mutation-induced specific changes in connexin (channel) functions and phenotypes of mutation-induced deafness. This lack of correspondence suggests that mutation-induced hearing loss is not directly determined by the mutation-induced pathogenic changes in channel function. Other factors, such as compensation and cooperation of other co-expressed connexins and *in vivo* functional impairment, may also play important roles in hearing loss.

## Connexin Expression and Function in the Cochlea

Connexin expression and gap junctional function in the inner ear have been extensively summarized by previous reviews [e.g., Zhao et al. (2006)]. Here, we briefly summarize main findings and information relevant to this review.

### Connexin expression in the cochlea

**Figure 1:**
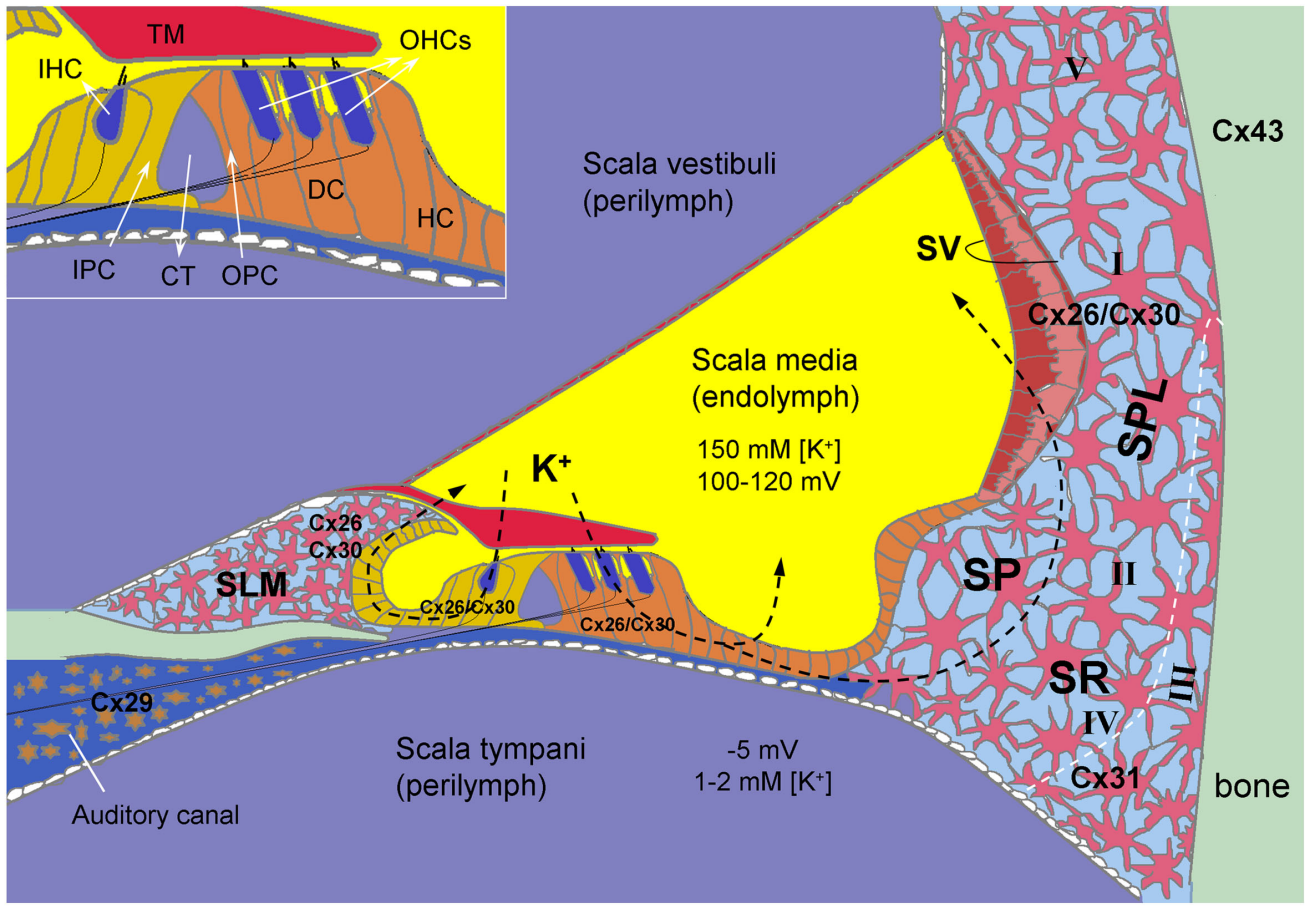
**Connexin expression and hypothesized K^+^-recycling in the cochlea**. Cx26 and Cx30 co-localized in supporting cells of the organ of Corti, the spiral limbus (SLM), the stria vascularis (SV), and fibrocytes of the spiral ligament (SPL). Cx31 is localized at type II and IV fibrocytes in the subcentral region (SR) below the spiral prominence (SP). Cx43 is expressed in the bone of the otic capsule. Cx29 is localized only at the Schwann cells wrapping the spiral ganglion neurons in the auditory canal. However, hair cells have no connexin expression. Inset: the organ of Corti. CT, cochlear tunnel; DC, Deiters cell; HC, Hensen cell; IHC, inner hair cell; IPC, inner pillar cell; OHCs, outer hair cells; OPC, outer pillar cell; TM, tectorial membrane; I–V, type I–V fibrocytes. Modified from Forge et al. (2003), Cohen-Salmon et al. (2004), Zhao and Yu (2006), and Liu and Zhao (2008).

In the cochlea, gap junctions exist extensively in the supporting cells in the organ of Corti, the stria vascularis (SV), the spiral ligament, the spiral limbus, and other cochlear non-sensory cells and structures (Kikuchi et al., 1995; Forge et al., 2003; Zhao and Yu, 2006; Liu and Zhao, 2008) (Figure 1). Two independent gap junctional networks have been identified in the inner ear: the epithelial gap junctional network between supporting cells in the auditory sensory epithelium in the organ of Corti and the connective tissue gap junctional network between the connective tissue cells in the cochlear lateral wall (Kikuchi et al., 1995). However, there is no connexin expression in hair cells (Kikuchi et al., 1995; Zhao and Santos-Sacchi, 1999; Zhao and Yu, 2006) or gap junctional coupling between outer hair cells (OHCs) and supporting cells (Yu and Zhao, 2009) (Figure 1).

Multiple connexin genes, *GJB2* (Cx26), *GJB6* (Cx30), *GJB3* (Cx31), *GJC3* (Cx29), and *GJA1* (Cx43), have been identified in the cochlea (Figure 1). Cx26 and Cx30 are the predominant isoforms and are widely expressed in the epithelial and connective tissues in a cell-specific and spatiotemporally complex fashion (Kikuchi et al., 1995; Lautermann et al., 1998; Forge et al., 2003; Zhao and Yu, 2006; Liu and Zhao, 2008). They are largely co-localized and can form homotypic and heterotypic/heteromeric gap junction channels between native cochlear supporting cells with asymmetrical-rectified gating, which allows one-directional passage (Zhao, 2000; Zhao and Santos-Sacchi, 2000).

Compared to the extensive expression of Cx26 and Cx30, the expression of other connexins in the cochlea is limited (Figure 1). Cx31 has been detected in type III fibrocytes in the spiral ligament in the cochlear lateral wall (Xia et al., 2000; López-Bigas et al., 2002; Forge et al., 2003). Cx29 in the cochlea is expressed on the Schwann cells wrapping the spiral ganglion (SG) neurons (Yang et al., 2005; Eiberger et al., 2006; Tang et al., 2006). Using immunofluorescent staining, Cx43 (*GJA1*) was initially reported on bone of the otic capsule and on cells lining the inside of the bony wall in adult animals (Forge et al., 2003; Suzuki et al., 2003). However, using *lacZ* reporter gene in Cx43 KO mice, it was found that Cx43 was highly expressed in the connective tissues and weakly expressed in the immature sensory epithelium of the cochlea from embryonic day 15.5 to the first week after birth and was almost exclusively expressed in the bone of the otic capsule after P8 (Cohen-Salmon et al., 2004).

### Gap junctional function in the cochlea

The hypothetical functions of gap junctions in the cochlea include K^+^-recycling (Santos-Sacchi and Dallos, 1983; Santos-Sacchi, 1987, 1991; Kikuchi et al., 1995; Spicer and Schulte, 1998; Zhao and Santos-Sacchi, 1998; Zhao, 2000) (Figure 1), nutrient and energy supply (Zhao, 2005; Zhao et al., 2005), intercellular signaling (Beltramello et al., 2005; Zhang et al., 2005; Zhao et al., 2005; Gossman and Zhao, 2008), endocochlear potential (EP) generation (Teubner et al., 2003; Chen et al., 2014), generation and maintenance of the unique electrochemical environments of the endolymph and perilymph (Cohen-Salmon et al., 2002; Teubner et al., 2003; Chen et al., 2014), and participation in active cochlear amplification (Yu and Zhao, 2009; Zhu et al., 2013, 2015). Recently, it has been reported that gap junction-mediated intercellular communication also plays an important role in epithelial repair in the cochlea (Forge et al., 2013; Jagger et al., 2014). Thus, dysfunction of gap junctions or connexin mutations can influence many aspects of cochlear function. For more detailed information, see Sections “Pathological Changes in the Cochlea in Cx26 Deficient Mice,” “Deafness Mechanisms Underlying Cx26 Deficiency Induced Hearing Loss,” and “Hearing Loss and Pathological Changes Induced by Mutations of Other Connexins.”

### Hemichannel function in the cochlea

Gap junction hemichannels can also function on the cell surface to provide an intracellular–extracellular conduit. Because hemichannels possess a relatively large pore size, they can release small molecules, such as ATP, which can subsequently activate purinergic P2 receptors to form an extracellular pathway for intercellular signaling. Gap junction hemichannels in the cochlear sensory epithelium can release ATP and IP_3_ and thus participate in intercellular signaling, control of OHC electromotility, K^+^-sinking, and gap junctional coupling (Zhao et al., 2005; Gossman and Zhao, 2008; Yu and Zhao, 2008; Zhu and Zhao, 2010, 2012). It has been reported that some deafness-associated mutants lose permeability to small biochemical molecules, thus impairing intercellular signaling (Beltramello et al., 2005; Zhang et al., 2005), or cause abnormal hemichannel activity eventually leading to cell lysis and death (Stong et al., 2006; Gerido et al., 2007). This indicates that gap junctional channels and connexin hemichannels may play an important role in cell signaling in the cochlea.

However, we recently found that Cx26 knockout or Cx30 knockout had little effect on ATP release in the cochlea under normal physiological conditions, while Panx1 deletion can abolish ATP release in the cochlea (Chen et al., 2015). These data suggest that pannexin channels rather than connexin hemichannels in the cochlea play an important role under normal physiological conditions.

### Connexin-specific function in the cochlea

Cx26 and Cx30 are predominant connexin isoforms in the cochlea (Forge et al., 2003; Zhao and Yu, 2006; Liu and Zhao, 2008). It has been found that inner ear gap junctions have strong charge selectivity; Cx26 is associated with anionic permeability in the cochlea (Zhao, 2005). This result is consistent with previous reports that Cx26 channels are permeable to both anionic and cationic molecules (Elfgang et al., 1995; Manthey et al., 2001; Beltramello et al., 2003), while Cx30 channels are impermeable to anionic molecules (Manthey et al., 2001; Beltramello et al., 2003). Thus, Cx26 in the cochlea is mainly responsible for permeability to anions and may play an important role in intercellular signaling, given that most cell signaling molecules (e.g., IP_3_, ATP, cAMP, and cGMP) are anions. This may be a reason why Cx26 but not Cx30 mutations induce high-incidence of hearing loss and why Cx30 knockin cannot restore hearing of Cx26 deficient mice (see Section “Hearing Restoration in Connexin Deficient Mice”).

However, functions of other connexins in the cochlea remain largely unclear. Cx29 is expressed on the Schwann cells at the auditory nerve and Cx43 is expressed in the bone. They may be involved in SG neuron activation and bone formation. Cx31 is mainly expressed in the type III fibrocytes in the cochlear lateral wall (Forge et al., 2003). Currently, little is known about Cx31 function in the cochlea, even though Cx31 mutations can also induce hearing loss (Liu et al., 2000; Oh et al., 2013; and also see Section “Hearing Loss and Pathological Changes Induced by Mutations of Other Connexins”).

## Pathological Changes in the Cochlea in Cx26 Deficient Mice

Cx26 and Cx30 are expressed in the cochlea and in the brain. However, cochlear implants can restore hearing function in connexin mutation-induced hearing loss (Rayess et al., 2015), indicating major pathological changes in the cochlea.

### Pathological changes in the cochlea in Cx26 knockout mice

Based on observations in Cx26 deletion mice, pathological changes of Cx26 deficiency in the cochlea include (i) cochlear developmental disorders, (ii) hair cell and SG neuron degeneration, (iii) EP reduction, and (iv) impairment in active cochlear amplification (Cohen-Salmon et al., 2002; Sun et al., 2009; Wang et al., 2009; Liang et al., 2012; Zhu et al., 2013, 2015; Chen et al., 2014).

#### Cochlear Developmental Disorders in Cx26 Deficient Mice

**Figure 2:**
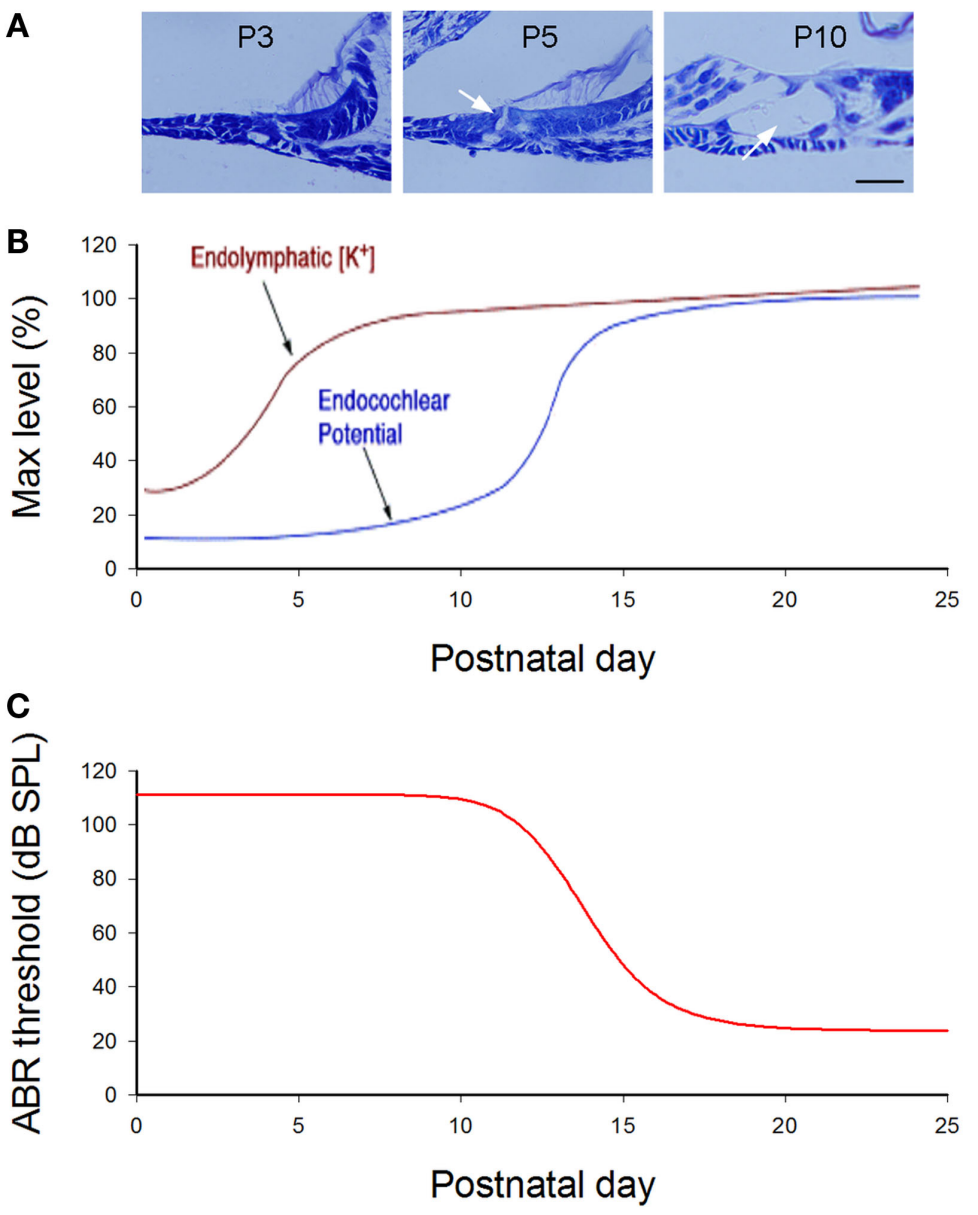
**Cochlear postnatal development and functional maturation in mice**. **(A)** Postnatal development of the cochlea. Arrows indicate that the cochlear tunnel starts to open at postnatal day 5 (P5) and fully opens at P10. **(B)** Postnatal developments of endocochlear potential (EP) and [K^+^]. Modified from Hibino et al. (2004). **(C)** Hearing maturation in mice. ABR thresholds dramatically drop at P11–16 and reach normal levels around P20. Modified from Liang et al. (2012).

**Figure 3:**
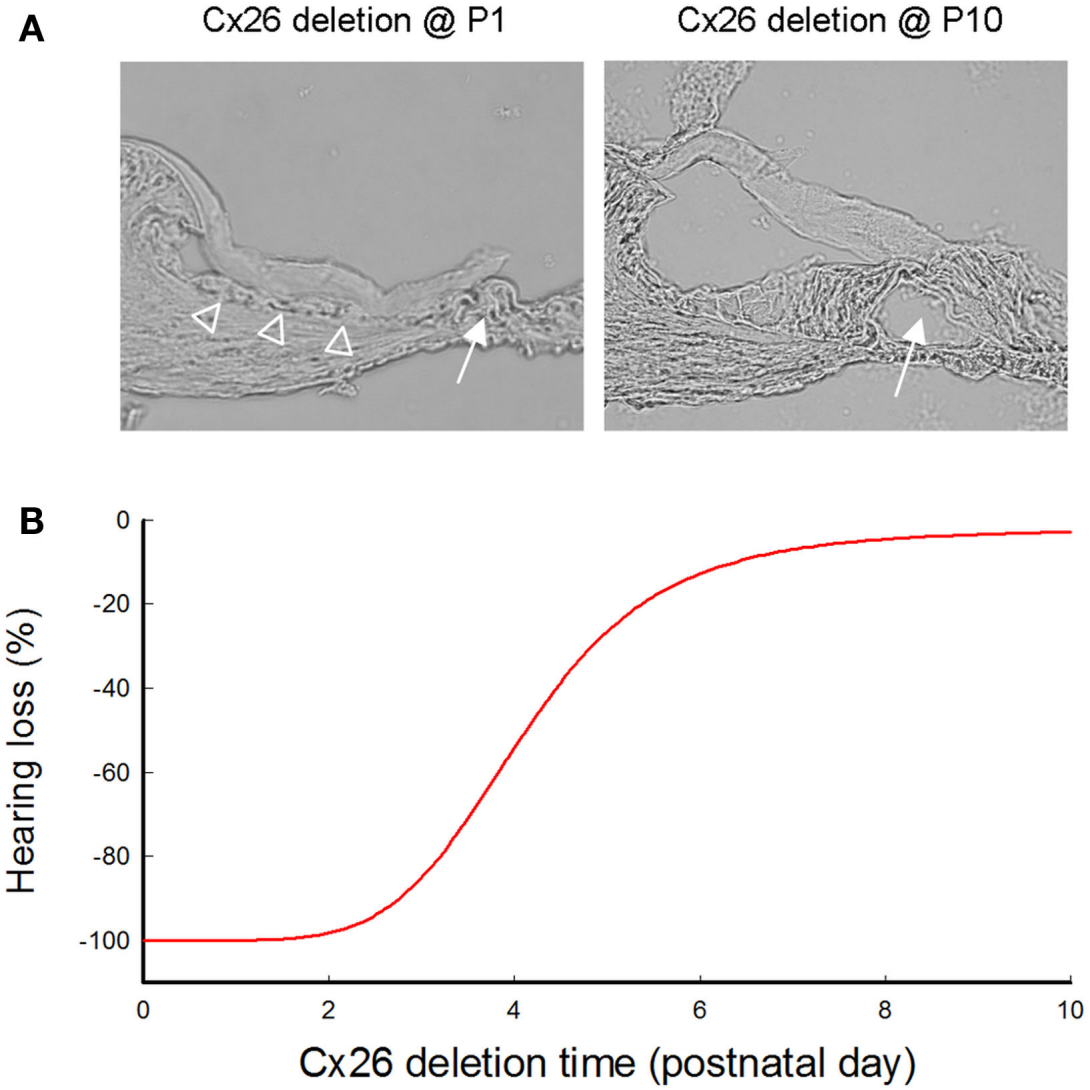
**Cochlear developmental disorders and hearing loss induced by deletion of Cx26 in the cochlea at different postnatal times**. **(A)** Cochlear development after deletion of Cx26 at P1 and P10. White arrows indicate that the cochlear tunnel is filled when Cx26 was deleted at P1 but developed normally when Cx26 was deleted at P10. Empty triangles indicate lack of the under-tectorial-membrane space; the tectorial membrane is attached to the inner sulcus cells following deletion of Cx26 at P1. **(B)** Hearing loss following deletion of Cx26 at different postnatal times. The ABR thresholds were measured at P30 and were normalized to that in WT mice. Corresponding to cochlear developmental disorders, deletion of Cx26 before P5 can induce severe hearing loss. However, hearing remains normal in young mice following deletion of Cx26 after P6. Modified from Chen et al. (2014).

**Figure 4:**
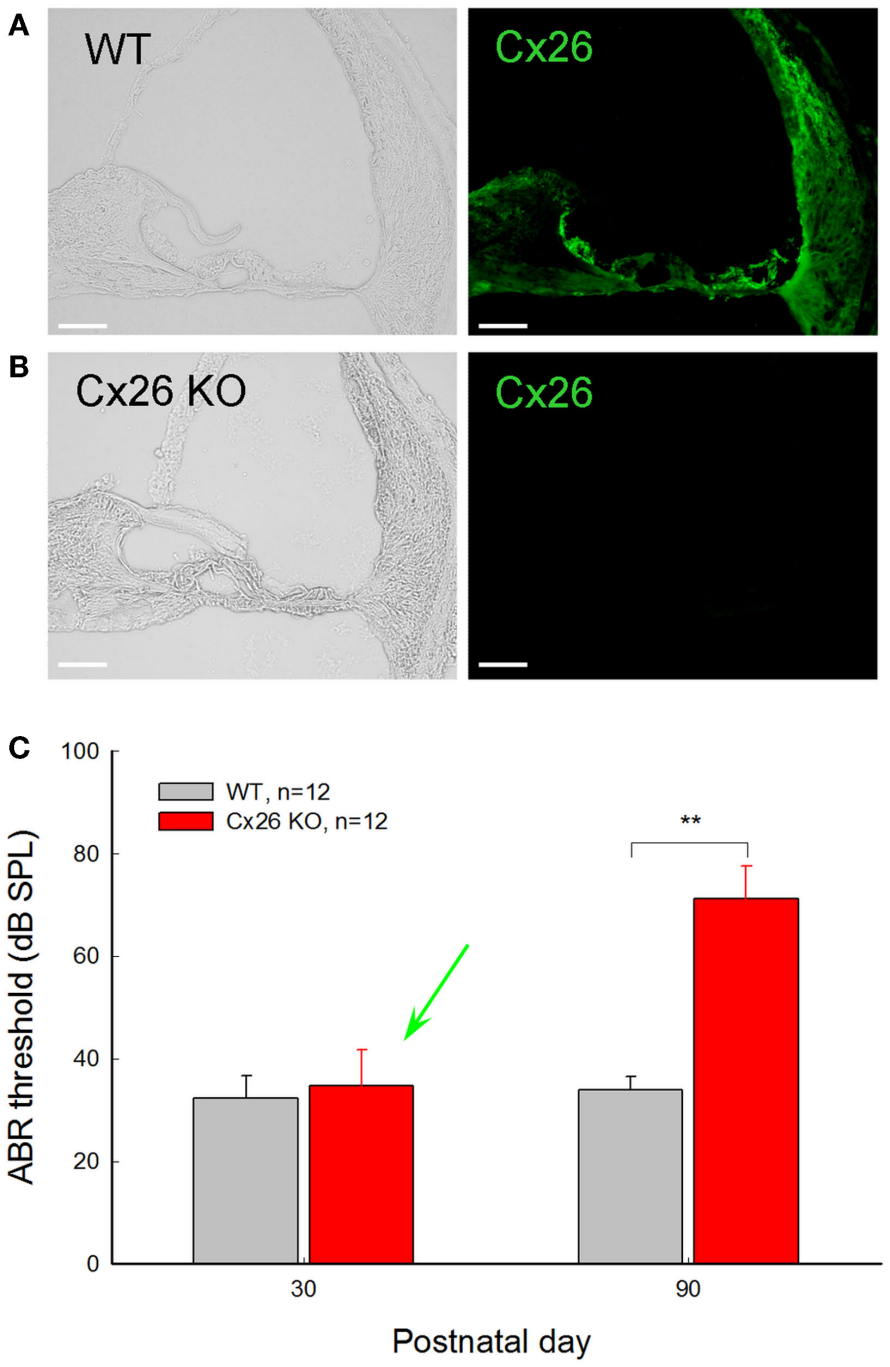
**Normal hearing in young Cx26 KO mice after deletion of Cx26 after birth**. **(A,B)** Immunofluorescent staining for Cx26 in the cochlea in WT and Cx26 KO mice at P30 following deletion of Cx26 at P10. No positive labeling is visible in the Cx26 KO mouse. **(C)** ABR thresholds of WT and Cx26 KO mice were measured at P30 and P90 following deletion of Cx26 at P10. A green arrow indicates that the ABR threshold in Cx26 KO mice at P30 remained normal, even though the expression of Cx26 in the cochlea was already deleted [see **(B)**]. At P90, Cx26 KO mice had a significant increase in ABR threshold, displaying hearing loss. **, *P* < 0.001, *t*-test. Scale bars: 50 μm. Modified from Zhu et al. (2015).

In mice, the cochlea shows normal morphology and the cochlear tunnel starts to open at postnatal day 5 (P5). By P10, the EP and K^+^ concentration in the endolymph rise to high levels (Ehret, 1976; Kraus and Aulbach-Kraus, 1981). Hearing starts around P14 and matures at P19–20 (Liang et al., 2012) (Figure 2). Cx26-null mice are embryonic lethal due to deficiency of transplacental uptake of glucose (Gabriel et al., 1998). Using conditional knockout techniques, it has been found that deletion of Cx26 in the cochlea at the embryonic period or P0–1 can cause cochlear developmental disorders (Wang et al., 2009; Liang et al., 2012; Chen et al., 2014). The tectorial membrane attaches at the inner sulcus cells and the under-tectorial-membrane space is lost; the cochlear tunnel is also filled (Figure 3A). Recently, we further found that deletion of Cx26 expression in the cochlea before P5 can induce cochlear developmental disorders with congenital deafness (Chen et al., 2014). However, with deletion of Cx26 after P5, cochlear development proceeded normally, the cochlear tunnel opened normally, and hearing remained normal at young ages (Chen et al., 2014) (Figures 3 and 4). These data suggest that Cx26 expression in the cochlea at the early postnatal development stage (<P5) is critical for cochlear postnatal development and maturation.

#### Cochlear Cell Degeneration in Cx26 Deficient Mice

Cx26 deletion in the cochlea can cause hair cell and SG neuron degeneration (Sun et al., 2009; Liang et al., 2012), even though hair cells have neither connexin expression (Kikuchi, et al., 1995; Zhao and Yu, 2006) nor gap junctional coupling (Zhao and Santos-Sacchi, 1999; Yu and Zhao, 2009). It has been reported that cell degeneration is detectable around P14 (Sun et al., 2009; Wang et al., 2009). However, substantial hair cell loss is not visible until adulthood. Severe cochlear hair cell loss and SG neuron degeneration also only occurred in middle and basal turns, i.e., in middle and high frequency regions (Sun et al., 2009; Wang et al., 2009; Liang et al., 2012). In addition, functional analyses show that hair cells in Cx26 deficient mice developed normally and retained normal function (Liang et al., 2012). So far, the mechanism underlying hair cell and SG neuron degeneration in Cx26 deficient mice remains unclear and needs to be further studied.

#### EP Reduction in Cx26 Deficient Mice

Endocochlear potential is a positive voltage (+100–120 mV) in the cochlear endolymph in the scala media (Figure 1) and is a driving force for propelling K^+^ ions through transduction channels in hair cells and producing auditory receptor current and potential. Positive EP is required for normal hearing and is generated in the cochlear lateral wall by a complex process (Chen and Zhao, 2014; Chen et al., 2015). Based on a “two-cell” model, EP generation is initiated at fibrocyte cells in the spiral ligament, where Na^+^/K^+^-ATPases and Na^+^, K^+^, 2Cl^-^ cotransporters depolarize cells to approximately -5 mV. Then, the intermediate cells in the SV are consequently depolarized to approximately -5 mV through gap junctional coupling, which is formed by Cx26 and Cx30 (Liu and Zhao, 2008). Since the apical membrane of the intermediate cells have ATP-dependent Kir4.1 K^+^ channels, this will generate a 110–120 mV transmembrane potential (Nernst’s K^+^ equilibrium potential) between the intracellular space and the intrastrial space, i.e., +115–125 mV in the intrastrial space with respect to normal extracellular space. Finally, this positive intrastrial potential eventually leads to positive EP (+100–120 mV) in the endolymph in the scala media (Chen and Zhao, 2014; Chen et al., 2015).

Gap junctional coupling is required for positive EP generation. Deletion of Cx26 expression in the cochlea can cause EP reduction (Cohen-Salmon et al., 2002; Chen et al., 2014; Zhu et al., 2015). In comparison with that of WT mice, EP in Cx26 KO mice was reduced by ~50% (Cohen-Salmon et al., 2002; Chen et al., 2014). However, EP remained normal following targeted-deletion of Cx26 expression in Deiters supporting cells and outer pillar supporting cells (Zhu et al., 2013), which are located around OHCs in the cochlear sensory epithelium (Figure 1). This indicates that impairment of the cochlear sensory gap junction network may have little effect on EP generation in the inner ear (Zhu et al., 2013; Chen and Zhao, 2014; Chen et al., 2015).

#### Reduction of Active Cochlear Amplification in Cx26 Deficient Mice

Normal hearing relies on active cochlear amplification to increase hearing sensitivity and frequency selectivity (Dallos, 2008; Hudspeth, 2008). Two forms of active cochlear mechanics have been proposed: one is prestin-based OHC electromotility; another is stereocilium-based hair bundle movement. OHC electromotility serves as a major source of active cochlear amplification in mammals (Brownell et al., 1985; Zheng et al., 2000; Ashmore, 2008). We found that Cx26 deficiency in the cochlear supporting cells can affect OHC electromotility (Yu and Zhao, 2009; Zhu et al., 2013, 2015), even though OHCs lack connexin expression and gap junctional coupling (Kikuchi, et al., 1995; Zhao and Santos-Sacchi, 1999; Zhao and Yu, 2006; Yu and Zhao, 2009). OHCs in Cx26 deficient mice still retain normal development and electromotility (Liang et al., 2012; Zhu et al., 2013, 2015). However, OHC electromotility was shifted (Zhu et al., 2013, 2015) and active cochlear amplification as measured by distortion product otoacoustic emission (DPOAE) was reduced (Zhu et al., 2013, 2015). Currently, detailed mechanisms for how connexin deficiency in the cochlear supporting cells influences OHC electromotility and eventually reduces active cochlear amplification remain unclear. This needs to be further examined in future studies.

### Hearing loss and pathological changes in the cochlea in Cx26 deafness mutation knockin mice

Currently, more than 100 Cx26 deafness mutations have been identified. However, only two Cx26 mutation knockin mouse models have been established. One is Cx26 p.R75W mutation knockin mouse line (Kudo et al., 2003), and another is Cx26 p.S17F knockin mouse line (Schütz et al., 2011).

Cx26 p.R75W knockin mice are viable. Similar to Cx26 knockout mice, Cx26 p.R75W transgenic mice displayed congenital deafness and cochlear developmental disorders, including filling of the cochlear tunnel (Kudo et al., 2003). Cell degeneration is also visible (Kudo et al., 2003; Inoshita et al., 2008). However, OHCs developed normally and retained normal function, but DPOAE was reduced (Inoshita et al., 2008; Minekawa et al., 2009).

For Cx26 p.S17F transgenic mice, homozygous mice are not viable, whereas the surviving heterozygous mice show a moderate hearing loss. The auditory brainstem response (ABR) threshold was increased by ~35 dB sound pressure level (SPL) in these mice, and EP was reduced by 20–40% (Schütz et al., 2011).

It should be noted that both p.R75W and p.S17F mutations are dominant mutations and cause syndromic hearing loss (Richard et al., 1998, 2002). Functional analyses showed that p.R75W mutation can target to the plasma membrane forming gap junctional plaques between cells, but has no channels function (Chen et al., 2005; Zhang et al., 2011; Kamiya et al., 2014), while p.S17F mutation cannot target to the plasma membrane forming functional gap junction channels or hemichannels (Richard et al., 2002; Lee et al., 2009a). These data further demonstrate that mutation-induced pathological changes in connexin (channel) function are not directly linked to the final auditory phenotypes. These data also suggest that deafness mechanism(s) underlying connexin mutation-induced syndromic and non-syndromic hearing loss may be similar.

## Deafness Mechanisms Underlying Cx26 Deficiency Induced Hearing Loss

Deletion of Cx26 in the cochlea can cause cochlear developmental disorders, severe hair cell loss, SG neuron degeneration, and EP reduction (Cohen-Salmon et al., 2002; Kudo et al., 2003; Sun et al., 2009; Wang et al., 2009; Liang et al., 2012; Chen et al., 2014). These findings provide invaluable information about pathological changes induced by Cx26 deficiency. However, the underlying deafness mechanism(s) is still unclear. Several deafness mechanisms, such as K^+^-recycling and Ca^++^-wave propagation hypotheses, have been proposed (Kelsell et al., 1997; Beltramello et al., 2005; Zhao et al., 2006). However, Cx26 mutations can cause various hearing loss phenotypes ranging from congenital deafness to late-onset, progressive hearing loss. Apparently, they have different underlying deafness mechanisms (Liang et al., 2012; Zhu et al., 2013, 2015; Chen et al., 2014).

### Hypothesized deafness mechanism for Cx26 deficiency induced hearing loss

#### Hypothesized K^+^-Recycling Impairment: Not a Principal Deafness Mechanism

During acoustic stimulation, K^+^ ions in the endolymph in the scala media flow into hair cells through the mechano-transduction channels to generate auditory receptor current and potential. Then, K^+^ ions are pumped out to the extracellular space in the perilymph through the hair cell’s lateral wall to restore hair cell function. The expelled K^+^ ions are subsequently sunken by cochlear supporting cells and are eventually transported back to the endolymph in the scala media via gap junctional pathways (Figure 1). This hypothesized function of gap junctional coupling in the inner ear was proposed about 30 years ago (Santos-Sacchi and Dallos, 1983; Kikuchi et al., 1995). Later, it was further hypothesized that Cx26 mutations may impair gap junctional coupling and disrupt such K^+^-recycling leading to K^+^ accumulation in the extracellular space near hair cells, thereby eventually damaging hair cells and causing hearing loss (Kelsell et al., 1997; Zhao et al., 2006). However, this hypothesized deafness mechanism lacks direct experimental evidence, even though it has been widely referred to. As mentioned above, not all Cx26 deafness mutations disrupt permeability of gap junction channels to ions; some deafness mutants can retain permeability to ions. Moreover, Cx26 mutations can cause various phenotypes of hearing loss (Castillo and Castillo, 2011; Chan and Chang, 2014), indicating that there are different underlying deafness mechanisms. Most importantly, our recent study demonstrates that mice with deletion of Cx26 in the cochlea after birth can retain normal hearing at young ages (Chen et al., 2014; Zhu et al., 2015) (Figure 4). These new data indicate that K^+^-recycling may not be important for hearing, if Cx26 deficiency disrupts the K^+^-recycling. Or, alternatively, the K^+^-recycling is important for hearing but not impaired by Cx26 deletion due to compensation by co-expressed Cx30. In any case, the hypothesized K^+^-recycling disruption cannot be a principal deafness mechanism for Cx26 deficiency induced hearing loss (Zhu et al., 2015).

#### Hypothesis of Impairment in Propagation of Ca^++^-Waves in the Cochlear Sensory Epithelium

As intercellular channels, gap junctions also play an important role in intercellular signaling and propagation of Ca^++^-waves among cells. The spread of Ca^++^-waves among cells can be enacted by the passage of Ca^++^ and IP_3_ between cells through gap junctions, or by the passage of ATP and IP_3_ through hemichannels and the subsequent activation of P2 purinergic receptors and IP_3_ receptors (Harris, 2001; Gossman and Zhao, 2008). Because the Cx26 deafness mutant p.R75W can impair the spread of Ca^++^-waves among cells (Beltramello et al., 2005; Yum et al., 2010; Zhang et al., 2011; Kamiya et al., 2014), it has been hypothesized that Cx26 deficiency may impair Ca^++^-wave propagation in the cochlea leading to hearing loss (Beltramello et al., 2005). However, it is currently unclear how such Ca^++^-wave propagation in the cochlea is related to hearing function and deafness, or whether it physiologically occurs in the cochlea. Moreover, it is unclear whether other deafness mutations can impair such Ca^++^-wave propagation, since some mutants can still form functional gap junctional channels permeable to ions (see above). Finally, similar to the K^+^-recycling disruption hypothesis, our recent finding that after deletion of Cx26 in the cochlea mice can still retain normal hearing at young ages (Chen et al., 2014; Zhu et al., 2015) (Figure 4) indicates that Ca^++^-wave propagation impairment cannot be a principal deafness mechanism for Cx26 deficiency induced hearing loss.

### Deafness mechanisms underlying Cx26 deficiency induced congenital deafness

As mentioned above, mouse models show that deletion of Cx26 at birth can cause congenital deafness with cochlear developmental disorders, hair cell degeneration, and EP reduction (Wang et al., 2009; Liang et al., 2012; Chen et al., 2014).

#### Cell degeneration is Not a Primary Cause for Cx26 Deficiency Induced Congenital Deafness

In mice, hearing starts at P14 and matures at P19–20. We (Liang et al., 2012) found that hearing in Cx26 KO mice is completely absent throughout the whole postnatal developmental period. The threshold of ABR, which is an auditory evoked potential in the brain recorded via electrodes placed on the scalp, was even greater than 110 dB SPL, indicating complete hearing loss. However, substantial hair cell loss was not visible until adulthood, although it has been reported that cell degeneration is detectable at young ages (Sun et al., 2009; Wang et al., 2009). Moreover, substantial cell degeneration was only visible at the middle and basal turns, i.e., in middle and high frequency regions (Sun et al., 2009; Wang et al., 2009; Liang et al., 2012). However, congenital, complete hearing loss occurred throughout the whole frequency range (Liang et al., 2012). Thus, hair cell loss is not a primary cause for Cx26 deficiency induced congenital deafness.

#### Congenital Deafness is Not Caused by EP Reduction

Endocochlear potential is also reduced in Cx26 KO mice (Cohen-Salmon et al., 2002; Chen et al., 2014). However, EP reduction is not associated with hearing loss in Cx26 KO mice. As mentioned above, the ABR threshold in Cx26 KO mice was even greater than 110 dB SPL, demonstrating complete hearing loss (Liang et al., 2012; Chen et al., 2014). However, the EP in Cx26 KO mice was not completely abolished and had a large variability. In some cases, the EP remained even at higher levels (>70 mV; Cohen-Salmon et al., 2002; Chen et al., 2014).

#### Cochlear Developmental Disorders are Associated with Congenital Deafness Generation

The congenital deafness in Cx26 KO mice, however, is associated with cochlear developmental disorders (Liang et al., 2012; Chen et al., 2014) (Figure 3). Deletion of Cx26 in the cochlea before P5 could cause cochlear developmental disorders, the cochlear tunnel was filled, and mice had congenital deafness (Chen et al., 2014). However, when Cx26 was deleted after P5, the cochlea displayed normal development, the cochlear tunnel was open as normal, and there was no congenital deafness (Chen et al., 2014; Zhu et al., 2015). These data suggest that congenital deafness induced by Cx26 deficiency is likely to result from cochlear developmental disorders (Figure 3). However, the detailed mechanisms for how Cx26 deficiency induces cochlear developmental disorders remain unclear.

### Deafness mechanisms underlying Cx26 deficiency induced late-onset hearing loss

Cx26 deficiency can also induce late-onset hearing loss. Using an inducible gene knockout technique, we found that deletion of Cx26 expression in the cochlea after P5 can induce late-onset, progressive hearing loss (Chen et al., 2014; Zhu et al., 2015). Mice retained normal hearing before P30. Then, hearing loss became apparent and severe at high frequencies (Zhu et al., 2015). This progression of hearing loss is similar to late-onset, progressive hearing loss observed in DFNA3 and DFNB1 non-syndromic deafness patients, who have normal hearing in early life, followed by hearing loss starting in childhood (Orzan and Murgia, 2007; Pollak et al., 2007; Gopalarao et al., 2008).

This Cx26 conditional KO mouse shows normal cochlear development and has no substantial hair cell loss (Zhu et al., 2015). EP was significantly reduced. However, the EP reduction was not associated with progressive hearing loss (Zhu et al., 2015), indicating that the EP reduction is also not a determining factor in Cx26 deficiency induced late-onset hearing loss. However, consistent with DPOAE reduction observed in patients (Engel-Yeger et al., 2002, 2003; Santarelli et al., 2007), the mouse model demonstrated a progressive DPOAE reduction, severe at high frequencies; functional analysis also showed that OHC electromotility was shifted (Zhu et al., 2015). These data indicate that late-onset hearing loss induced by Cx26 deficiency may result from impairment in active cochlear amplification. However, the underlying cellular mechanism remains undetermined.

## Hearing Loss and Pathological Changes Induced by Mutations of Other Connexins

In addition to *GJB2* (Cx26), multiple connexin genes *GJB6* (Cx30), *GJB3* (Cx31), *GJC3* (Cx29), and *GJA1* (Cx43), have been identified in the cochlea. Mutations of these connexin genes can also cause hearing loss, even if they are rare.

### Cx30 mutation-induced hearing loss and pathological changes

Cx30 is a predominant connexin isoform, which is extensively co-expressed with Cx26 in the cochlea (Forge et al., 2003; Zhao and Yu, 2006; Liu and Zhao, 2008). Cx30 mutations can also induce hearing loss. It has been reported that a 342-kb deletion truncating the *GJB6* (Cx30) gene is associated with non-syndromic hearing loss through either homozygous deletion of Cx30 or digenic inheritance of a Cx30 deletion and a Cx26 mutation (Castillo et al., 2002). It has also been reported that the Cx30 missense mutation p.T5M at the N-terminus is associated with autosomal dominant non-syndromic deafness (DFNA3), characterized by late-onset, middle to high frequency hearing loss (Grifa et al., 1999). Functional analysis shows that the Cx30 p.T5M mutation can track to the plasma membrane but defective channel activity was observed in dye transfer assay (Common et al., 2002; Berger et al., 2014). The mutation can also impair permeability to IP_3_ (Zhang et al., 2005).

It has been found that Cx30 p.A40V and p.I248V can also cause non-syndromic hearing loss (Yang et al., 2007; Oh et al., 2013). Wang et al. (2011) reported that Cx30 p.A40V mutation could not target to the plasma membrane and accumulated in the Golgi body. The mutant also exerted a dominant negative effect on both WT Cx30 and Cx26, which impaired gap junction formation. However, a recent report (Oh et al., 2013) showed that both p.A40V and p.I248V deafness mutations could target to the plasma membrane to form gap junctional channels; they were permeable to calcium ions, but reduced permeability to propidium iodide dye.

Cx30^T5M/T5M^ knockin mice exhibited a mild, but significant increase in their hearing thresholds of about 15 dB at all frequencies (Schütz et al., 2010). Western blot analysis showed significantly downregulated expression levels of Cx26 and Cx30. However, Cx26 and Cx30 retained normal distribution patterns. The cochlea and EP also developed normally. Electrical coupling, probed by dual patch-clamp recordings, was normal. However, transfer of the fluorescent tracer calcein between cochlear non-sensory cells was reduced (Schütz et al., 2010).

Cx30 knockout mice also show deafness with absence of EP (Teubner et al., 2003; Chen et al., 2014). After P18, the cochlear sensory epithelium starts to degenerate via cell apoptosis (Sun et al., 2009). However, it has been found that Cx26 expression at the protein level in this Cx30 KO mouse line also decreased to 30%. Interestingly, restoration of Cx26 expression in this Cx30 KO mouse line by knockin of an external Cx26 copy can rescue its hearing (Ahmad et al., 2007). Recently, it has been reported that a new Cx30 conditional KO mouse line, in which half of Cx26 expression is preserved, displays normal hearing (Boulay et al., 2013). These data imply that hearing loss in Cx30 KO mice may result from the accompanied reduction in Cx26 expression. Currently, it is still unclear whether sole Cx30 deletion can induce hearing loss.

### Cx31 mutation-induced hearing loss and pathological changes

The Cx31 mutations can cause both recessive and dominant non-syndromic hearing loss, characterized by late-onset moderate deafness affecting high frequencies (Liu et al., 2000; Oh et al., 2013). Functional analyses showed that some deafness mutations (e.g., p.V27M, p.V43M, and p.V84I) could target to the plasma membrane to form gap junctional plaques but lost permeability to dyes and ions (Oh et al., 2013). It has also been reported that deafness mutation Cx31 p.V174M cannot target to the plasma membrane and accumulated in the lysosomes in the mutant-transfected HeLa cells (Li et al., 2014). The mutant could also impair Cx26 WT intracellular trafficking to the plasma membrane, but did not influence trafficking of Cx31 WT (Li et al., 2014). Cx31 deficiency in mice causes transient placental dysmorphogenesis but does not impair hearing (Plum et al., 2001).

### Cx29 mutation-induced hearing loss and pathological changes

Cx29 mutations can also cause non-syndromic hearing loss (Hong et al., 2010a; Wang et al., 2010). Cx29 KO could result in delay in maturation of hearing threshold, severe loss of myelination, a prolongation in latency and distortion in the wave I of the ABR, loss of high frequency sensitivity, and increased sensitivity to noise damage (Tang et al., 2006). However, a study from another group showed that deletion of Cx29 had no effect on hearing (Eiberger et al., 2006). The reason for these conflicting results is currently unclear.

### Cx43 mutation-induced hearing loss and pathological changes

Cx43 (*GJA1*) mutations were also initially linked to non-syndromic autosomal recessive deafness (Liu et al., 2001). However, it is now clear that the mutations are located in the *GJA1* pseudogene rather than in *GJA1* (Paznekas et al., 2003; Hong et al., 2010b). Recently, a new study (Kim et al., 2013) reported that immunofluorescent staining for Cx43 showed strong labeling in the mid-internal auditory canal in the modiolus, which represents the transition zone between Schwann cells and oligodendrocytes. Young Cx43 heterozygous mice (3–4 months old) showed mild-moderate hearing loss (Kim et al., 2013). The hearing loss became severe when the mice were old.

## Connexin Expression and Pathogenesis in the Human Cochlea

Despite extensive studies of connexin expression in the animal cochlea (Kikuchi et al., 1995; Forge et al., 2003; Zhao and Yu, 2006; Liu and Zhao, 2008), little is known about connexin expression in the human inner ear. It has been reported that Cx26 and Cx30 in the human cochlea have similar expression patterns to animals (Liu et al., 2009). Recently, by use of the human fetal cochlea, it has been found that the expression of Cx26 and Cx30 is detectable in the outer sulcus cells, but not in the spiral ligament in the cochlear lateral wall at 18 weeks of gestation (Locher et al., 2015). However, because of the very limited availability of samples, connexin expression in the human cochlea still remains largely undetermined.

Moreover, although there are numerous clinical reports of connexin mutation-associated hearing loss, there is little description of the pathological changes in humans. Studies of human temporal bone CT imaging showed that approximately 50% of ears of subjects with *GJB2* mutations have temporal bone anomaly (Propst et al., 2006; Lee et al., 2009b). Few studies on the pathogenesis of Cx26 mutations have been done. So far, there is only one case-report of a patient that had Cx26 p.G35delG mutation and p.E101G missense mutation with profound hearing loss (Jun et al., 2000). Microscopic observation revealed nearly complete degeneration of hair cells and agenesis of the SV but no neural degeneration. However, other factors, such as aging, cannot be excluded as contributors to these pathological changes. Further studies are needed to define the mutation-associated pathological changes in the human cochlea.

## Hearing Restoration in Connexin Deficient Mice

It has been reported that restoration of Cx26 expression by knockin of an external Cx26 copy in the Cx30 KO mouse line can rescue its hearing, because the Cx26 expression level in the Cx30 KO mice was also dramatically reduced (Ahmad et al., 2007). However, Cx30 expression in Cx26 deficient mice was not reduced (Zhu et al., 2015), and knockin of an external Cx30 copy in Cx26 deficient mice cannot rescue hearing (Qu et al., 2012). Unlike Cx30 knockin, it has been found that knockin of Cx32 (*GJB1*) in Cx26 deficient mice can restore hearing (Degen et al., 2011). As mentioned above (Section “Connexin-Specific Function in the Cochlea”), permeability of gap junctions and hemichannels in the cochlea is highly charge dependent and Cx26 expression is associated with anionic permeability in the cochlea (Zhao, 2005). Cx26 and Cx30 are predominant connexin isoforms in the cochlea (Forge et al., 2003; Zhao and Yu, 2006; Liu and Zhao, 2008). However, Cx26 channels are permeable to both anionic and cationic molecules (Elfgang et al., 1995; Manthey et al., 2001; Beltramello et al., 2003), while Cx30 channels are impermeable to anionic molecules (Manthey et al., 2001; Beltramello et al., 2003). Thus, Cx26 is able to compensate for Cx30 function, while Cx30 cannot completely compensate for Cx26 function. Cx32 is similar to Cx26 in that it is permeable to both cationic and anionic molecules (Elfgang et al., 1995). This may be a reason why Cx32 knockin but not Cx30 knockin can restore hearing function in Cx26 deficient mice. However, the detailed mechanisms need to be further clarified.

Recently, it has also been reported that a genetic approach to restore Cx26 expression in the cochlea in Cx26 KO mice after birth can preserve both hair cells and SG neurons but cannot restore hearing function (Yu et al., 2014). This indicates that restoration of hearing function is dependent on not only the level of gene expression but also the timing of gene expression.

## Summary and Prospection

Cx26 mutations are the major cause of hereditary deafness and can induce congenital deafness and late-onset hearing loss (Castillo and Castillo, 2011; Chan and Chang, 2014). Connexin-deletion mouse models show that congenital deafness induced by Cx26 deficiency is associated with cochlear developmental disorders rather than hair cell loss and EP reduction (Liang et al., 2012; Chen et al., 2014), and that late-onset hearing loss induced by Cx26 deficiency results from the reduction of active cochlear amplification (Zhu et al., 2013, 2015). The new data also demonstrate that the hypothesized K^+^-recycling disruption is not a principal deafness mechanism for Cx26 deficiency induced hearing loss (Chen et al., 2014; Zhu et al., 2015). These new studies provide a full image of deafness mechanisms for Cx26 mutation-induced hearing loss and also provide important information for developing new protective and therapeutic strategies for this common deafness.

However, the cellular mechanisms underlying Cx26 deficiency induced cochlear developmental disorders remain unclear. The mechanisms whereby Cx26 deficiency in the supporting cells influences OHC electromotility and active cochlear amplification are also unclear. Moreover, it is unknown whether specific mutations of connexins will produce the same pathological changes as connexin knockout mice. Finally, little is known about pathological changes in the human cochlea, and it is unclear whether the observed pathological changes in mice are similar to humans.

Creation of specific connexin mutation knockin mouse models and investigation of pathological changes in the human cochlea are urgently required. The knockin transgenic mice can also provide important information about the relationship between mutated protein expression and absence of protein expression *in vivo*. Recently, it has been reported that administration of vitamins A, C, and E and magnesium ameliorated progressive hearing loss in a child with Cx26 mutation (Thatcher et al., 2014). However, the underlying mechanism(s) is not known. These new findings further indicate that a more comprehensive understanding of this common hereditary deafness is important and urgent and will have a tremendous impact on many people, given connexin mutations account for more than 50% of hereditary hearing loss.

## Conflict of Interest Statement

The authors declare that the research was conducted in the absence of any commercial or financial relationships that could be construed as a potential conflict of interest.

## References

[B1] Ahmad S. Tang W. Chang Q. Qu Y. Hibshman J. Li Y. (2007). Restoration of connexin26 protein level in the cochlea completely rescues hearing in a mouse model of human connexin30-linked deafness. Proc. Natl. Acad. Sci. U.S.A. 104, 1337–1341. 10.1073/pnas.0606855104 17227867 PMC1783143

[B2] Ashmore J. (2008). Cochlear outer hair cell motility. Physiol. Rev. 88, 173–210. 10.1152/physrev.00044.2006 18195086

[B3] Baranova A. Ivanov D. Petrash N. Pestova A. Skoblov M. Kelmanson I. (2004). The mammalian pannexin family is homologous to the invertebrate innexin gap junction proteins. Genomics 83, 706–716. 10.1016/j.ygeno.2003.09.025 15028292

[B4] Beltramello M. Bicego M. Piazza V. Ciubotaru C. D. Mammano F. D’Andrea P. (2003). Permeability and gating properties of human connexins 26 and 30 expressed in HeLa cells. Biochem. Biophys. Res. Commun. 305, 1024–1033. 10.1016/S0006-291X(03)00868-4 12767933

[B5] Beltramello M. Piazza V. Bukauskas F. F. Pozzan T. Mammano F. (2005). Impaired permeability to Ins(1,4,5)P3 in a mutant connexin underlies recessive hereditary deafness. Nat. Cell Biol. 7, 63–69. 10.1038/ncb1205 15592461

[B6] Bennett M. V. L. Barrio L. C. Bargiello T. A. Spray D. C. Hertzberg E. Saez J. C. (1991). Gap junctions: new tools, new answers, new questions. Neuron 6, 305–320. 10.1016/0896-6273(91)90241-Q 1848077

[B7] Bennett M. V. L. Contreras J. E. Bukauskas F. F. Saez J. C. (2003). New roles for astrocytes: gap junction hemichannels have something to communicate. Trends Neurosci. 26, 610–617. 10.1016/j.tins.2003.09.008 14585601 PMC3694339

[B8] Berger A. C. Kelly J. J. Lajoie P. Shao Q. Laird D. W. (2014). Mutations in Cx30 that are linked to skin disease and non-syndromic hearing loss exhibit several distinct cellular pathologies. J. Cell Sci. 127, 1751–1764. 10.1242/jcs.138230 24522190

[B9] Bicego M. Beltramello M. Melchionda S. Carella M. Piazza V. Zelante L. (2006). Pathogenetic role of the deafness-related M34T mutation of Cx26. Hum. Mol. Genet. 15, 2569–2587. 10.1093/hmg/ddl184 16849369 PMC2829448

[B10] Boassa D. Ambrosi C. Qiu F. Dahl G. Gaietta G. Sosinsky G. (2007). Pannexin1 channels contain a glycosylation site that targets the hexamer to the plasma membrane. J. Biol. Chem. 282, 31733–31743. 10.1074/jbc.M702422200 17715132

[B11] Boulay A. C. del Castillo F. J. Giraudet F. Hamard G. Giaume C. Petit C. (2013). Hearing is normal without connexin30. J. Neurosci. 33, 430–434. 10.1523/JNEUROSCI.4240-12.2013 23303923 PMC6704917

[B12] Brownell W. E. Bader C. R. Bertrand D. Ribaupierre Y. (1985). Evoked mechanical responses of isolated cochlear outer hair cells. Science 227, 194–196. 10.1126/science.3966153 3966153

[B13] Bruzzone R. Hormuzdi S. G. Barbe M. T. Herb A. Monyer H. (2003a). Pannexins, a family of gap junction proteins expressed in brain. Proc. Natl. Acad. Sci. U.S.A. 100, 13644–13649. 10.1073/pnas.2233464100 14597722 PMC263867

[B14] Bruzzone R. Veronesi V. Gomes D. Bicego M. Duval N. Marlin S. (2003b). Loss-of-function and residual channel activity of connexin26 mutations associated with non-syndromic deafness. FEBS Lett. 533, 79–88. 10.1016/S0014-5793(02)03755-9 12505163

[B15] Castillo F. J. Castillo I. (2011). The DFNB1 subtype of autosomal recessive non-syndromic hearing impairment. Front. Biosci. 17:3252–3274. 10.2741/3910 21622233

[B16] Castillo I. Villamar M. Moreno-Pelayo M. A. del Castillo F. J. Alvarez A. Tellería D. (2002). A deletion involving the connexin 30 gene in nonsyndromic hearing impairment. N. Engl. J. Med. 346, 243–249. 10.1056/NEJMoa012052 11807148

[B17] Chan D. K. Chang K. W. (2014). GJB2-associated hearing loss: systematic review of worldwide prevalence, genotype, and auditory phenotype. Laryngoscope 124, E34–E53. 10.1002/lary.24332 23900770

[B18] Chen J. Chen J. Zhu Y. Liang C. Zhao H. B. (2014). Deafness induced by connexin 26 (GJB2) deficiency is not determined by endocochlear potential (EP) reduction but is associated with cochlear developmental disorders. Biochem. Biophys. Res. Commun. 448, 28–32. 10.1016/j.bbrc.2014.04.016 24732355 PMC4105360

[B19] Chen J. Zhao H. B. (2014). The role of an inwardly rectifying K^+^ channel (Kir4.1) in the inner ear and hearing loss. Neuroscience 265, 137–146. 10.1016/j.neuroscience.2014.01.036 24480364 PMC4007161

[B20] Chen J. Zhu Y. Liang C. Chen J. Zhao H. B. (2015). Pannexin1 channels dominate ATP release in the cochlea ensuring endocochlear potential and auditory receptor potential generation and hearing. Sci. Rep. 5, 10762 10.1038/srep10762 PMC4451810 26035172

[B21] Chen Y. Deng Y. Bao X. Reuss L. Altenberg G. A. (2005). Mechanism of the defect in gap-junctional communication by expression of a connexin 26 mutant associated with dominant deafness. FASEB J. 19, 1516–1518. 10.1096/fj.04-3491fje 16009703

[B22] Choung Y. H. Moon S. K. Park H. J. (2002). Functional study of GJB2 in hereditary hearing loss. Laryngoscope 112, 1667–1671. 10.1097/00005537-200209000-00026 12352684

[B23] Cohen-Salmon M. Maxeiner S. Krüger O. Theis M. Willecke K. Petit C. (2004). Expression of the connexin43- and connexin45-encoding genes in the developing and mature mouse inner ear. Cell Tissue Res. 316, 15–22. 10.1007/s00441-004-0861-2 14986102

[B24] Cohen-Salmon M. Ott T. Michel V. Hardelin J. P. Perfettini I. Eybalin M. (2002). Targeted ablation of connexin26 in the inner ear epithelial gap junction network causes hearing impairment and cell death. Curr. Biol. 12, 1106–1111. 10.1016/S0960-9822(02)00904-1 12121617 PMC4030438

[B25] Common J. E. Becker D. Di W. L. Leigh I. M. O’Toole E. A. Kelsell D. P. (2002). Functional studies of human skin disease- and deafness-associated connexin 30 mutations. Biochem. Biophys. Res. Commun. 298, 651–656. 10.1016/S0006-291X(02)02517-2 12419304

[B26] Dallos P. (2008). Cochlear amplification, outer hair cells and prestin. Curr. Opin. Neurobiol. 18, 370–376. 10.1016/j.conb.2008.08.016 18809494 PMC2630119

[B27] D’Andrea P. Veronesi V. Bicego M. Melchionda S. Zelante L. Di Iorio E. (2002). Hearing loss: frequency and functional studies of the most common connexin26 alleles. Biochem. Biophys. Res. Commun. 296, 685–691. 10.1016/S0006-291X(02)00891-4 12176036

[B28] de Zwart-Storm E. A. Hamm H. Stoevesandt J. Steijlen P. M. Martin P. E. van Geel M. (2008). A novel missense mutation in GJB2 disturbs gap junction protein transport and causes focal palmoplantar keratoderma with deafness. J. Med. Genet. 45, 161–166. 10.1136/jmg.2007.052332 17993581

[B29] Degen J. Schütz M. Dicke N. Strenzke N. Jokwitz M. Moser T. (2011). Connexin32 can restore hearing in connexin26 deficient mice. Eur. J. Cell Biol. 90, 817–824. 10.1016/j.ejcb.2011.05.001 21813206

[B30] Deng Y. Chen Y. Reuss L. Altenberg G. A. (2006). Mutations of connexin 26 at position 75 and dominant deafness: essential role of arginine for the generation of functional gap-junctional channels. Hear. Res. 220, 87–94. 10.1016/j.heares.2006.07.004 16945493

[B31] Denoyelle F. Lina-Granade G. Plauchu H. Bruzzone R. Chaïb H. Lévi-Acobas F. (1998). Connexin26 gene linked to a dominant deafness. Nature 393, 319–320. 10.1038/30639 9620796

[B32] Ehret G. (1976). Development of absolute auditory thresholds in the house mouse (*Mus musculus*). J. Am. Audiol. Soc. 1, 179–184. 956003

[B33] Eiberger J. Kibschull M. Strenzke N. Schober A. Büssow H. Wessig C. (2006). Expression pattern and functional characterization of connexin29 in transgenic mice. Glia 53, 601–611. 10.1002/glia.20315 16435366

[B34] Elfgang C. Eckert R. Lichtenberg-Fraté H. Butterweck A. Traub O. Klein R. A. (1995). Specific permeability and selective formation of gap junction channels in connexin-transfected HeLa cells. J. Cell Biol. 129, 805–817. 10.1083/jcb.129.3.805 7537274 PMC2120441

[B35] Engel-Yeger B. Zaaroura S. Zlotogora J. Shalev S. Hujeirat Y. Carrasquillo M. (2002). The effects of a connexin 26 mutation – 35delG – on oto-acoustic emissions and brainstem evoked potentials: homozygotes and carriers. Hear. Res. 163, 93–100. 10.1016/S0378-5955(01)00386-0 11788203

[B36] Engel-Yeger B. Zaaroura S. Zlotogora J. Shalev S. Hujeirat Y. Carrasquillo M. (2003). Otoacoustic emissions and brainstem evoked potentials in compound carriers of connexin 26 mutations. Hear. Res. 175, 140–151. 10.1016/S0378-5955(02)00719-0 12527132

[B37] Estivill X. Fortina P. Surrey S. Rabionet R. Melchionda S. D’Agruma L. (1998). Connexin-26 mutations in sporadic and inherited sensorineural deafness. Lancet 351, 394–398. 10.1016/S0140-6736(97)11124-2 9482292

[B38] Forge A. Becker D. Casalotti S. Edwards J. Marziano N. Nevill G. (2003). Gap junctions in the inner ear: comparison of distribution patterns in different vertebrates and assessment of connexin composition in mammals. J. Comp. Neurol. 467, 207–231. 10.1002/cne.10916 14595769

[B39] Forge A. Jagger D. J. Kelly J. J. Taylor R. R. (2013). Connexin30-mediated intercellular communication plays an essential role in epithelial repair in the cochlea. J. Cell Sci. 126, 1703–1712. 10.1242/jcs.125476 23424196

[B40] Gabriel H. D. Jung D. Bützler C. Temme A. Traub O. Winterhager E. (1998). Transplacental uptake of glucose is decreased in embryonic lethal connexin26-deficient mice. J. Cell Biol. 140, 1453–1461. 10.1083/jcb.140.6.1453 9508777 PMC2132681

[B41] Gerido D. A. DeRosa A. M. Richard G. White T. W. (2007). Aberrant hemichannel properties of Cx26 mutations causing skin disease and deafness. Am. J. Physiol. Cell Physiol. 293, C337–C345. 10.1152/ajpcell.00626.2006 17428836

[B42] Goodenough D. A. Paul D. L. (2003). Beyond the gap: functions of unpaired connexon channels. Nat. Rev. Mol. Cell Biol. 4, 284–294. 10.1038/nrm1072 12671651

[B43] Gopalarao D. Kimberling W. J. Jesteadt W. Kelley P. M. Beauchaine K. L. Cohn E. S. (2008). Is hearing loss due to mutations in the connexin 26 gene progressive? Int. J. Audiol. 47, 11–20. 10.1080/14992020701602087 18196482

[B44] Gossman D. G. Zhao H. B. (2008). Hemichannel-mediated inositol 1,4,5-trisphosphate (IP_3_) release in the cochlea: a novel mechanism of IP_3_ intercellular signaling. Cell Commun. Adhes. 15, 305–315. 10.1080/15419060802357217 18979296 PMC5543712

[B45] Grifa A. Wagner C. A. D’Ambrosio L. Melchionda S. Bernardi F. Lopez-Bigas N. (1999). Mutations in GJB6 cause nonsyndromic autosomal dominant deafness at DFNA3 locus. Nat. Genet. 23, 16–18. 10.1038/12612 10471490

[B46] Harris A. L. (2001). Emerging issues of connexin channels: biophysics fills the gap. Q. Rev. Biophys. 34, 325–472. 10.1017/S0033583501003705 11838236

[B47] Hibino H. Higashi-Shingai K. Fujita A. Iwai K. Ishii M. Kurachi Y. (2004). Expression of an inwardly rectifying K^+^ channel, Kir5.1, in specific types of fibrocytes in the cochlear lateral wall suggests its functional importance in the establishment of endocochlear potential. Eur. J. Neurosci. 19, 76–84. 10.1111/j.1460-9568.2004.03092.x 14750965

[B48] Hong H. M. Yang J. J. Su C. C. Chang J. Y. Li T. C. Li S. Y. (2010a). A novel mutation in the connexin 29 gene may contribute to nonsyndromic hearing loss. Hum. Genet. 127, 191–199. 10.1007/s00439-009-0758-y 19876648

[B49] Hong H. M. Yang J. J. Shieh J. C. Lin M. L. Li S. Y. (2010b). Novel mutations in the connexin43 (GJA1) and GJA1 pseudogene may contribute to nonsyndromic hearing loss. Hum. Genet. 127, 545–551. 10.1007/s00439-010-0791-x 20130915

[B50] Hudspeth A. J. (2008). Making an effort to listen: mechanical amplification in the ear. Neuron 59, 530–545. 10.1016/j.neuron.2008.07.012 18760690 PMC2724262

[B51] Inoshita A. Iizuka T. Okamura H. O. Minekawa A. Kojima K. Furukawa M. (2008). Postnatal development of the organ of corti in dominant-negative Gjb2 transgenic mice. Neuroscience 156, 1039–1047. 10.1016/j.neuroscience.2008.08.027 18793701

[B52] Jagger D. J. Nickel R. Forge A. (2014). Gap junctional coupling is essential for epithelial repair in the avian cochlea. J. Neurosci. 34, 15851–15860. 10.1523/JNEUROSCI.1932-14.2014 25429127 PMC4244460

[B53] Jun A. I. McGuirt W. T. Hinojosa R. Green G. E. Fischel-Ghodsian N. Smith R. J. (2000). Temporal bone histopathology in connexin 26-related hearing loss. Laryngoscope 110, 269–275. 10.1097/00005537-200002010-00016 10680928

[B54] Kamiya K. Yum S. W. Kurebayashi N. Muraki M. Ogawa K. Karasawa K. (2014). Assembly of the cochlear gap junction macromolecular complex requires connexin 26. J. Clin. Invest. 124, 1598–1607. 10.1172/JCI67621 24590285 PMC3973107

[B55] Kelley P. M. Harris D. J. Comer B. C. Askew J. W. Fowler T. Smith S. D. (1998). Novel mutations in the connexin 26 gene (GJB2) that cause autosomal recessive (DFNB1) hearing loss. Am. J. Hum. Genet. 62, 792–799. 10.1086/301807 9529365 PMC1377046

[B56] Kelsell D. P. Dunlop J. Stevens H. P. Lench N. J. Liang J. N. Parry G. (1997). Connexin 26 mutations in hereditary non-syndromic sensorineural deafness. Nature 387, 80–83. 10.1038/387080a0 9139825

[B57] Kikuchi T. Kimura R. S. Paul D. L. Adams J. C. (1995). Gap junctions in the rat cochlea: immunohistochemical and ultrastructural analysis. Anat. Embryol. 191, 101–118. 10.1007/BF00186783 7726389

[B58] Kim A. H. Nahm E. Sollas A. Mattiace L. Rozental R. (2013). Connexin 43 and hearing: possible implications for retrocochlear auditory processing. Laryngoscope 123, 3185–3193. 10.1002/lary.24249 23817980

[B59] Kraus H. J. Aulbach-Kraus K. (1981). Morphological changes in the cochlea of the mouse after the onset of hearing. Hear. Res. 4, 89–102. 10.1016/0378-5955(81)90038-1 7204263

[B60] Kudo T. Kure S. Ikeda K. Xia A. P. Katori Y. Suzuki M. (2003). Transgenic expression of a dominant-negative connexin26 causes degeneration of the organ of corti and non-syndromic deafness. Hum. Mol. Genet. 12, 995–1004. 10.1093/hmg/ddg116 12700168

[B61] Lautermann J. ten Cate W. J. F. Altenhoff P. Grümmer R. Traub O. Frank H. G. (1998). Expression of the gap-junction connexins 26 and 30 in the rat cochlea. Cell Tissue Res. 294, 415–420. 10.1007/s004410051192 9799458

[B62] Lee J. R. Derosa A. M. White T. W. (2009a). Connexin mutations causing skin disease and deafness increase hemichannel activity and cell death when expressed in *Xenopus* oocytes. J. Invest. Dermatol. 129, 870–878. 10.1038/jid.2008.335 18987669 PMC6463483

[B63] Lee K. H. Larson D. A. Shott G. Rasmussen B. Cohen A. P. Benton C. (2009b). Audiologic and temporal bone imaging findings in patients with sensorineural hearing loss and GJB2 mutations. Laryngoscope 119, 554–558. 10.1002/lary.20162 19235794 PMC7065710

[B64] Li T. C. Kuan Y. H. Ko T. Y. Li C. Yang J. J. (2014). Mechanism of a novel missense mutation, p.V174M, of the human connexin31 (GJB3) in causing nonsyndromic hearing loss. Biochem. Cell Biol. 92, 251–257. 10.1139/bcb-2013-0126 24913888

[B65] Liang C. Zhu Y. Zong L. Liu G. J. Zhao H. B. (2012). Cell degeneration is not a primary cause for Cx26 deficiency associated hearing loss. Neurosci. Lett. 528, 36–41. 10.1016/j.neulet.2012.08.085 22975134 PMC3467974

[B66] Liu W. Boström M. Kinnefors A. Rask-Andersen H. (2009). Unique expression of connexins in the human cochlea. Hear. Res. 250, 55–62. 10.1016/j.heares.2009.01.010 19450429

[B67] Liu X. Z. Xia X. J. Adams J. Chen Z. Y. Welch K. O. Tekin M. (2001). Mutations in GJA1 (connexin 43) are associated with non-syndromic autosomal recessive deafness. Hum. Mol. Genet. 10, 2945–2951. 10.1093/hmg/10.25.2945 11741837

[B68] Liu X. Z. Xia X. J. Xu L. R. Pandya A. Liang C. Y. Blanton S. H. (2000). Mutations in connexin31 underlie recessive as well as dominant non-syndromic hearing loss. Hum. Mol. Genet. 9, 63–67. 10.1093/hmg/9.1.63 10587579

[B69] Liu Y. P. Zhao H. B. (2008). Cellular characterization of connexin26 and connexin30 expression in the cochlear lateral wall. Cell Tissue Res. 333, 395–403. 10.1007/s00441-008-0641-5 18581144 PMC2548271

[B70] Locher H. de Groot J. C. van Iperen L. Huisman M. A. Frijns J. H. Chuva de Sousa Lopes S. M. (2015). Development of the stria vascularis and potassium regulation in the human fetal cochlea: insights into hereditary sensorineural hearing loss. Dev. Neurobiol. 10.1002/dneu.22279 25663387 PMC5024031

[B71] López-Bigas N. Arbonés M. L. Estivill X. Simonneau L. (2002). Expression profiles of the connexin genes, Gjb1 and Gjb3, in the developing mouse cochlea. Mech. Dev. 119(Suppl. 1), S111–S115. 10.1016/S0925-4773(03)00102-3 14516671

[B72] Mani R. S. Ganapathy A. Jalvi R. Srikumari Srisailapathy C. R. Malhotra V. Chadha S. (2009). Functional consequences of novel connexin 26 mutations associated with hereditary hearing loss. Eur. J. Hum. Genet. 17, 502–509. 10.1038/ejhg.2008.179 18941476 PMC2986212

[B73] Manthey D. Banach K. Desplantez T. Lee C. G. Kozak C. A. Traub O. (2001). Intracellular domains of mouse connexin26 and -30 affect diffusional and electrical properties of gap junction channels. J. Membr. Biol. 181, 137–148. 10.1007/s00232-001-0017-1 11420600

[B74] Martin P. E. Coleman S. L. Casalotti S. O. Forge A. Evans W. H. (1999). Properties of connexin26 gap junctional proteins derived from mutations associated with non-syndromal hereditary deafness. Hum. Mol. Genet. 8, 2369–2376. 10.1093/hmg/8.13.2369 10556284

[B75] Minekawa A. Abe T. Inoshita A. Iizuka T. Kakehata S. Narui Y. (2009). Cochlear outer hair cells in a dominant-negative connexin26 mutant mouse preserve non-linear capacitance in spite of impaired distortion product otoacoustic emission. Neuroscience 164, 1312–1319. 10.1016/j.neuroscience.2009.08.043 19712724

[B76] Oh S. K. Choi S. Y. Yu S. H. Lee K. Y. Hong J. H. Hur S. W. (2013). Evaluation of the pathogenicity of GJB3 and GJB6 variants associated with nonsyndromic hearing loss. Biochim. Biophys. Acta 1832, 285–291. 10.1016/j.bbadis.2012.05.009 22617145

[B77] Orzan E. Murgia A. (2007). Connexin 26 deafness is not always congenital. Int. J. Pediatr. Otorhinolaryngol. 71, 501–507. 10.1016/j.ijporl.2006.12.002 17222463

[B78] Palmada M. Schmalisch K. Böhmer C. Schug N. Pfister M. Lang F. (2006). Loss of function mutations of the GJB2 gene detected in patients with DFNB1-associated hearing impairment. Neurobiol. Dis. 22, 112–118. 10.1016/j.nbd.2005.10.005 16300957

[B79] Paznekas W. A. Boyadjiev S. A. Shapiro R. E. Daniels O. Wollnik B. Keegan C. E. (2003). Connexin 43 (GJA1) mutations cause the pleiotropic phenotype of oculodentodigital dysplasia. Am. J. Hum. Genet. 72, 408–418. 10.1086/346090 12457340 PMC379233

[B80] Penuela S. Bhalla R. Gong X. Q. Cowan K. N. Celetti S. J. Cowan B. J. (2007). Pannexin 1 and pannexin 3 are glycoproteins that exhibit many distinct characteristics from the connexin family of gap junction proteins. J. Cell Sci. 120, 3772–3783. 10.1242/jcs.009514 17925379

[B81] Plum A. Winterhager E. Pesch J. Lautermann J. Hallas G. Rosentreter B. (2001). Connexin31-deficiency in mice causes transient placental dysmorphogenesis but does not impair hearing and skin differentiation. Dev. Biol. 231, 334–347. 10.1006/dbio.2000.0148 11237463

[B82] Pollak A. Skórka A. Mueller-Malesińska M. Kostrzewa G. Kisiel B. Waligóra J. (2007). M34T and V37I mutations in GJB2 associated hearing impairment: evidence for pathogenicity and reduced penetrance. Am. J. Med. Genet. A 143A, 2534–2543. 10.1002/ajmg.a.31982 17935238

[B83] Propst E. J. Blaser S. Stockley T. L. Harrison R. V. Gordon K. A. Papsin B. C. (2006). Temporal bone imaging in GJB2 deafness. Laryngoscope 116, 2178–2186. 10.1097/01.mlg.0000244389.68568.a7 17146393

[B84] Qu Y. Tang W. Zhou B. Ahmad S. Chang Q. Li X. (2012). Early developmental expression of connexin26 in the cochlea contributes to its dominate functional role in the cochlear gap junctions. Biochem. Biophys. Res. Commun. 417, 245–250. 10.1016/j.bbrc.2011.11.093 22142852 PMC3259187

[B85] Rabionet R. Zelante L. López-Bigas N. D’Agruma L. Melchionda S. Restagno G. (2000). Molecular basis of childhood deafness resulting from mutations in the GJB2 (connexin 26) gene. Hum. Genet. 106, 40–44. 10.1007/s004390051007 10982180

[B86] Rayess H. M. Weng C. Murray G. S. Megerian C. A. Semaan M. T. (2015). Predictive factors and outcomes of cochlear implantation in patients with connexin 26 mutation: a comparative study. Am. J. Otolaryngol. 36, 7–12. 10.1016/j.amjoto.2014.08.010 25270357

[B87] Richard G. Rouan F. Willoughby C. E. Brown N. Chung P. Ryynänen M. (2002). Missense mutations in GJB2 encoding connexin-26 cause the ectodermal dysplasia keratitis-ichthyosis-deafness syndrome. Am. J. Hum. Genet. 70, 1341–1348. 10.1086/339986 11912510 PMC447609

[B88] Richard G. White T. W. Smith L. E. Bailey R. A. Compton J. G. Paul D. L. (1998). Functional defects of Cx26 resulting from a heterozygous missense mutation in a family with dominant deaf-mutism and palmoplantar keratoderma. Hum. Genet. 103, 393–399. 10.1007/s004390050839 9856479

[B89] Santarelli R. Scimemi P. Dal Monte E. Genovese E. Arslan E. (2007). Auditory neuropathy in systemic sclerosis: a speech perception and evoked potential study before and after cochlear implantation. Eur. Arch. Otorhinolaryngol. 263, 809–815. 10.1007/s00405-006-0075-1 16763823

[B90] Santos-Sacchi J. (1987). Cell coupling differs in the in vitro and in vivo organ of corti. Hear. Res. 25, 227–232. 10.1016/0378-5955(87)90094-3 3558131

[B91] Santos-Sacchi J. (1991). Isolated supporting cells from the organ of corti: some whole cell electrical characteristics and estimates of gap junction conductance. Hear. Res. 52, 89–98. 10.1016/0378-5955(91)90190-K 2061216

[B92] Santos-Sacchi J. Dallos P. (1983). Intercellular communication in the supporting cells of the organ of corti. Hear. Res. 9, 317–326. 10.1016/0378-5955(83)90034-5 6841286

[B93] Schütz M. Auth T. Gehrt A. Bosen F. Körber I. Strenzke N. (2011). The connexin26 S17F mouse mutant represents a model for the human hereditary keratitis-ichthyosis-deafness syndrome. Hum. Mol. Genet. 20, 28–39. 10.1093/hmg/ddq429 20926451

[B94] Schütz M. Scimemi P. Majumder P. De Siati R. D. Crispino G. Rodriguez L. (2010). The human deafness-associated connexin 30 T5M mutation causes mild hearing loss and reduces biochemical coupling among cochlear non-sensory cells in knock-in mice. Hum. Mol. Genet. 19, 4759–4773. 10.1093/hmg/ddq402 20858605 PMC2989887

[B95] Skerrett I. M. Di W. L. Kasperek E. M. Kelsell D. P. Nicholson B. J. (2004). Aberrant gating, but a normal expression pattern, underlies the recessive phenotype of the deafness mutant connexin26M34T. FASEB J. 18, 860–862. 10.1096/fj.03-0763fje 15033936

[B96] Sosinsky G. E. Boassa D. Dermietzel R. Duffy H. S. Laird D. W. MacVicar B. A. (2011). Pannexin channels are not gap junction hemichannels. Channels (Austin) 5, 193–197. 10.4161/chan.5.3.15765 21532340 PMC3704572

[B97] Spicer S. S. Schulte B. A. (1998). Evidence for a medial K+ recycling pathway from inner hair cells. Hear. Res. 118, 1–12. 10.1016/S0378-5955(98)00006-9 9606057

[B98] Stong B. C. Chang Q. Ahmad S. Lin X. (2006). A novel mechanism for connexin 26 mutation linked deafness: cell death caused by leaky gap junction hemichannels. Laryngoscope 16, 2205–2210. 10.1097/01.mlg.0000241944.77192.d2 17146396

[B99] Sun Y. Tang W. Chang Q. Wang Y. Kong W. Lin X. (2009). Connexin30 null and conditional connexin26 null mice display distinct pattern and time course of cellular degeneration in the cochlea. J. Comp. Neurol. 516, 569–579. 10.1002/cne.22117 19673007 PMC2846422

[B100] Suzuki T. Takamatsu T. Oyamada M. (2003). Expression of gap junction protein connexin43 in the adult rat cochlea: comparison with connexin26. J. Histochem. Cytochem. 51, 903–912. 10.1177/002215540305100705 12810840

[B101] Tang W. Zhang Y. Chang Q. Ahmad S. Dahlke I. Yi H. (2006). Connexin29 is highly expressed in cochlear Schwann cells, and it is required for the normal development and function of the auditory nerve of mice. J. Neurosci. 26, 1991–1999. 10.1523/JNEUROSCI.5055-05.2006 16481432 PMC6674919

[B102] Teubner B. Michel V. Pesch J. Lautermann J. Cohen-Salmon M. Sohl G. (2003). Connexin30 (Gjb6)-deficiency causes severe hearing impairment and lack of endocochlear potential. Hum. Mol. Genet. 12, 13–21. 10.1093/hmg/ddg001 12490528

[B103] Thatcher A. Le Prell C. Miller J. Green G. (2014). ACEMg supplementation ameliorates progressive connexin 26 hearing loss in a child. Int. J. Pediatr. Otorhinolaryngol. 78, 563–565. 10.1016/j.ijporl.2013.12.030 24439969

[B104] Thomas T. Telford D. Laird D. W. (2004). Functional domain mapping and selective trans-dominant effects exhibited by Cx26 disease-causing mutations. J. Biol. Chem. 279, 19157–19168. 10.1074/jbc.M314117200 14978038

[B105] Thonnissen E. Rabionet R. Arbones M. L. Estivill X. Willecke K. Ott T. (2002). Human connexin26 (GJB2) deafness mutations affect the function of gap junction channels at different levels of protein expression. Hum. Genet. 111, 190–197. 10.1007/s00439-002-0750-2 12189493

[B106] Wang H. L. Chang W. T. Li A. H. Yeh T. H. Wu C. Y. Chen M. S. (2003). Functional analysis of connexin-26 mutants associated with hereditary recessive deafness. J. Neurochem. 84, 735–742. 10.1046/j.1471-4159.2003.01555.x 12562518

[B107] Wang W. H. Liu Y. F. Su C. C. Su M. C. Li S. Y. Yang J. J. (2011). A novel missense mutation in the connexin30 causes nonsyndromic hearing loss. PLoS ONE 6:e21473. 10.1371/journal.pone.0021473 21731760 PMC3123352

[B108] Wang W. H. Yang J. J. Lin Y. C. Yang J. T. Chan C. H. Li S. Y. (2010). Identification of novel variants in the Cx29 gene of nonsyndromic hearing loss patients using buccal cells and restriction fragment length polymorphism method. Audiol. Neurootol. 15, 81–87. 10.1159/000231633 19657183

[B109] Wang Y. Chang Q. Tang W. Sun Y. Zhou B. Li H. (2009). Targeted connexin26 ablation arrests postnatal development of the organ of corti. Biochem. Biophys. Res. Commun. 385, 33–37. 10.1016/j.bbrc.2009.05.023 19433060 PMC2713729

[B110] Willecke K. Eiberger J. Degen J. Eckardt D. Romualdi A. Guldenagel M. (2002). Structural and functional diversity of connexin genes in the mouse and human genome. J. Biol. Chem. 383, 725–737. 10.1515/BC.2002.076 12108537

[B111] Xia A. P. Ikeda K. Katori Y. Oshima T. Kikuchi T. Takasaka T. (2000). Expression of connexin 31 in the developing mouse cochlea. Neuroreport 11, 2449–2453. 10.1097/00001756-200008030-00022 10943702

[B112] Yang J. J. Huang S. H. Chou K. H. Liao P. J. Su C. C. Li S. Y. (2007). Identification of mutations in members of the connexin gene family as a cause of nonsyndromic deafness in Taiwan. Audiol. Neurootol. 12, 198–208. 10.1159/000099024 17259707

[B113] Yang J. J. Liao P. J. Su C. C. Li S. Y. (2005). Expression patterns of connexin 29 (GJE1) in mouse and rat cochlea. Biochem. Biophys. Res. Commun. 338, 723–728. 10.1016/j.bbrc.2005.09.193 16236250

[B114] Yu N. Zhao H. B. (2008). ATP activates P2x receptors and requires extracellular Ca^++^ participation to modify outer hair cell nonlinear capacitance. Pflugers Arch. 457, 453–461. 10.1007/s00424-008-0522-5 18491132 PMC5531446

[B115] Yu N. Zhao H. B. (2009). Modulation of outer hair cell electromotility by cochlear supporting cells and gap junctions. PLoS ONE 4:e7923. 10.1371/journal.pone.0007923 19936276 PMC2775161

[B116] Yu Q. Wang Y. Chang Q. Wang J. Gong S. Li H. (2014). Virally expressed connexin26 restores gap junction function in the cochlea of conditional Gjb2 knockout mice. Gene Ther. 21, 71–80. 10.1038/gt.2013.59 24225640 PMC3881370

[B117] Yum S. W. Zhang J. Scherer S. S. (2010). Dominant connexin26 mutants associated with human hearing loss have trans-dominant effects on connexin30. Neurobiol. Dis. 38, 226–236. 10.1016/j.nbd.2010.01.010 20096356 PMC2868926

[B118] Zelante L. Gasparini P. Estivill X. Melchionda S. D’Agruma L. Govea N. (1997). Connexin26 mutations associated with the most common form of non-­syndromic neurosensory autosomal recessive deafness (DFNB1) in Mediterraneans. Hum. Mol. Genet. 6, 1605–1609. 10.1093/hmg/6.9.1605 9285800

[B119] Zhang J. Scherer S. S. Yum S. W. (2011). Dominant Cx26 mutants associated with hearing loss have dominant-negative effects on wild type Cx26. Mol. Cell. Neurosci. 47, 71–78. 10.1016/j.mcn.2010.10.002 21040787 PMC3132585

[B120] Zhang Y. Tang W. Ahmad S. Sipp J. A. Chen P. Lin X. (2005). Gap junction-mediated intercellular biochemical coupling in cochlear supporting cells is required for normal cochlear functions. Proc. Natl. Acad. Sci. U.S.A. 102, 15201–15206. 10.1073/pnas.0501859102 16217030 PMC1257692

[B121] Zhao H. B. (2000). Directional rectification of gap junctional voltage gating between Deiters cells in the inner ear of guinea pig. Neurosci. Lett. 296, 105–108. 10.1016/S0304-3940(00)01626-8 11108992

[B122] Zhao H. B. (2005). Connexin26 is responsible for anionic molecule permeability in the cochlea for intercellular signaling and metabolic communications. Eur. J. Neurosci. 21, 1859–1868. 10.1111/j.1460-9568.2005.04031.x 15869481 PMC2548270

[B123] Zhao H. B. Kikuchi T. Ngezahayo A. White T. W. (2006). Gap junctions and cochlear homeostasis. J. Membr. Biol. 209, 177–186. 10.1007/s00232-005-0832-x 16773501 PMC1609193

[B124] Zhao H. B. Santos-Sacchi J. (1998). Effect of membrane tension on gap junctional conductance of supporting cells in corti’s organ. J. Gen. Physiol. 112, 447–455. 10.1085/jgp.112.4.447 9758863 PMC2229429

[B125] Zhao H. B. Santos-Sacchi J. (1999). Auditory collusion and a coupled couple of outer hair cells. Nature 399, 359–362. 10.1038/20686 10360573

[B126] Zhao H. B. Santos-Sacchi J. (2000). Voltage gating of gap junctions in cochlear supporting cells: evidence for nonhomotypic channels. J. Membr. Biol. 175, 17–24. 10.1007/s002320001051 10811964

[B127] Zhao H. B. Yu N. (2006). Distinct and gradient distributions of connexin26 and connexin30 in the cochlear sensory epithelium of guinea pigs. J. Comp. Neurol. 499, 506–518. 10.1002/cne.21113 16998915 PMC2553046

[B128] Zhao H. B. Yu N. Fleming C. R. (2005). Gap junctional hemichannel-mediated ATP release and hearing controls in the inner ear. Proc. Natl. Acad. Sci. U.S.A. 102, 18724–18729. 10.1073/pnas.0506481102 16344488 PMC1317927

[B129] Zheng J. Shen W. He D. Z. Long K. B. Madison L. D. Dallos P. (2000). Prestin is the motor protein of cochlear outer hair cells. Nature 405, 149–155. 10.1038/35012009 10821263

[B130] Zhu Y. Chen J. Liang C. Zong L. Chen J. Jones R. O. (2015). connexin26 (*GJB2*) deficiency reduces active cochlear amplification leading to late-onset hearing loss. Neuroscience 284, 719–729. 10.1016/j.neuroscience.2014.10.061 25451287 PMC4268423

[B131] Zhu Y. Liang C. Chen J. Zong L. Chen G. D. Zhao H. B. (2013). Active cochlear amplification is dependent on supporting cell gap junctions. Nat. Commun. 4, 1786. 10.1038/ncomms2806 23653198 PMC3675877

[B132] Zhu Y. Zhao H. B. (2010). ATP-mediated potassium recycling in the cochlear supporting cells. Purinergic Signal. 6, 221–229. 10.1007/s11302-010-9184-9 20806014 PMC2912999

[B133] Zhu Y. Zhao H. B. (2012). ATP activates P2X receptors to mediate gap junctional coupling in the cochlea. Biochem. Biophys. Res. Commun. 426, 528–532. 10.1016/j.bbrc.2012.08.119 22982314 PMC3471361

